# Improvement of medication adherence in adolescents and young adults with SLE using web-based education with and without a social media intervention, a pilot study

**DOI:** 10.1186/s12969-018-0232-2

**Published:** 2018-03-14

**Authors:** Lisabeth V. Scalzi, Christopher S. Hollenbeak, Emily Mascuilli, Nancy Olsen

**Affiliations:** Pennsylvania State University, MC A480; 90 Hope Drive, Hershey, PA 17033 USA

**Keywords:** Systemic lupus erythematosus, Feasibility, Self-management, Medication adherence, Social media

## Abstract

**Background:**

Self-management skills, including medication management, are vital to the health of adolescents and young adults with systemic lupus erythematosus (SLE). The purpose of this study was to assess the feasibility and preliminary effects of an online educational program in a cohort of adolescent and young adults with SLE with and without a social media (SM) experience.

**Methods:**

Adolescents and young adults with SLE participated weekly for 8 sessions on a web-based educational program about SLE created specifically for this project. Subjects were randomized to respond to questions at the end of each weekly module in a journal or on a SM forum with other SLE subjects. Patients were surveyed prior to initiating the study, (T0) and 6 weeks after completion of the sessions (T1). Medication adherence for hydroxychloroquine, utilizing the medication possession ratio (MPR), was compared for the 3 months preceding T0 and for the 3 months following T1.

**Results:**

Twenty-seven of the 37 subjects (73%) enrolled completed the study, including the two required sets of surveys. Reasons for being lost to follow up included being too busy, forgetting, and/or not seeing email reminders. Medication adherence improved in all subjects (*p* < 0.001). The percentage of the SM intervention group that was adherent (MPR ≥ 80%) significantly improved from 50% to 92% (*p* = 0.03), while the control group did not. Secondary outcome measures that improved, only in the SM group, included self-efficacy, sense of agency (SOA), sense of community (SOC), and empowerment. There was a strong correlation between empowerment with SOA and SOC and in turn a strong correlation with SOA and SOC with MPR, providing a possible explanation for why social media participation helped to improve medication adherence. Subjective reporting of medication adherence was not reliably correlated to MPR.

**Conclusions:**

This pilot study has demonstrated feasibility for the use of an online educational SLE website, recruitment, and measurement of chosen outcome measures. This study provides evidence for a larger multi-site trial which has the potential to address an important service gap by delivering self-management education and peer interactions in a format that is accessible, and engaging to young people with SLE.

**Trial registration:**

Trial registration: NCT03218033. Retrospectively registered 14 July 2017.

**Electronic supplementary material:**

The online version of this article (10.1186/s12969-018-0232-2) contains supplementary material, which is available to authorized users.

## Background

Learning to live with systemic lupus erythematosus (SLE) can be extremely difficult for children who are affected and for their parents. Self-management skills are vital to the physical and emotional health of these patients [1]. Self-management can be defined as “the individual’s ability to manage the symptoms, treatment, and physical and psychological consequences and life style changes inherent in living with chronic illness”. One of the key components of self-management is medication adherence. Non-adherence with medications in patients with systemic lupus has been noted to be as poor as 40% [2, 3]. Success in disease control can be significantly impacted by such non-adherence. Poor medication compliance is associated with higher SLE disease activity scores and higher SLE disease activity in turn has been demonstrated to be significantly associated with a decline in quality of life [4]. Much attention has been paid to how to improve self-management skills in adults, but less is known about how to target adolescents, an age group with a complex set of emotional and developmental needs.

The World Health Organization has described empowerment as a “prerequisite for health” and a “proactive partnership and patient self-care strategy to improve health outcomes and quality of life among the chronically ill” [5]. In the context of health care, empowerment refers to encouraging people to participate as equal and active partners in decisions about their disease. Patient empowerment aims to improve self-efficacy, promote lifestyle changes, and help to shift the disease management from health professionals to the individual. Self-efficacy theory refers to a person’s belief in his/her capacity to successfully modify his/her health care related behavior [6]. Web-based interventions can improve empowerment and the effects of these interventions may remain for prolonged periods of time [7]. The theoretical mechanism for improved empowerment using social media stems from the rewards of posting and reading posts. Posting activities build a sense of agency (SOA), while reading others’ posts build relatedness and a heightened sense of community (SOC). SOA is the feeling that one has a competent, confident, and assertive voice. SOC is the feeling that members 1) have a sense of belonging to a community, 2) matter to each other, and 3) have needs that will be met via a commitment to be with one another. Together, the SOA and SOC are known to influence a person’s sense of empowerment [8, 9].

Using the internet as a delivery system for information and support provides many advantages, especially for adolescents. The internet, including online forums, allows people with similar experiences and/or interests to interact. It is not limited by physical distance or schedules and allows adolescents to share information that they otherwise may not feel comfortable sharing in person. Effects of interactive health communication that have been demonstrated include improved disease knowledge, clinical outcomes, self-efficacy, and feelings of being socially supported [10]. This study examines the feasibility of recruitment for a trial examining the effect of participation in an online educational website for adolescents and young adults with SLE, with and without social media participation. The study also gathers pilot data to determine changes in secondary outcomes including medication adherence and potential associations of this improvement if present.

## Methods

### Educational website

A publically available website, www.facinglupustogether.com, [11] was created specifically for the purposes of this study. Content was written by authors LS and EM and images of disease manifestations shared, with permissions, from the American College of Rheumatology’s Rheumatology Image Library. The website contains eight modules that are titled,: 1) “Making the Transition and Taking charge of My Medications”, 2) “Learning About Lupus”, 3) “Learning About Lupus Medications”, 4) “Managing Symptoms of Lupus”, 5) “How do I Handle Lupus and My Family”, 6) “How I Handle Lupus and My Friends”, 7) “Lupus and Stress”, and 8) “My Personal Goals and How I Will Achieve Them”.

Cognitive pretesting was provided by 15 age-matched adolescents from the Penn State Day Program Adolescent Eating Disorder Program. The participants provided suggestions on readability, usability, website design, and topics that would be interesting to this age group. Participants were able to complete each module within 20 min.

### Subjects and enrollment

Inclusion criterion included 1) age between 13 and 23 at the time of recruitment, 2) having the diagnosis of SLE made or confirmed by a pediatric or adult rheumatologist at Penn State Children’s Hospital/Hershey Medical Center, 3) Having been diagnosed with SLE ≤ age of 16 years, and 4) having regular internet access. Exclusion criterion included 1) age < 13 or > 23 years, and 2) comorbid medical or psychiatric illness that would affect the outcome measures. In order to have an appropriate age distribution between groups, subjects were first identified to be in a younger (13–17 years) or older (18–23 years) age group. Members of the two age groups were then randomized consecutively either to the control group or the social media (SM) experimental group. All participants completed online Research Electronic Data Capture (REDCap) [12] surveys prior to starting the study (T0) and also 6 weeks after completing the 8-week study (T1). All outcome measures and subject information, except for the medication possession ratio, were measured via responses on the two electronic surveys at T0 and T1. For any participants who did not complete the assigned surveys, the research nurse reached out to them via email and/or telephone to complete the required information. This research protocol was approved by the Penn State Hershey Medical Center Institutional Review Board and all participants/ legal guardians signed written informed consent/assent.

### Study intervention

The intervention phase was 8 weeks in duration. Participants visited the Facinglupustogether.com website and participated in consecutive weekly online educational modules. At the end of each module there were open-ended questions pertaining to the subject of each module. The control group answered the questions in writing in the provided journals. The journals were then sent back to the research team after completion of the 8-week intervention. The SM group answered the questions on an online social media forum with other SM participants. SM participants were encouraged to provide feedback or questions about the material or personal questions that arose in response to each module. A pediatric rheumatologist monitored the SM site every day throughout the study period. Personal messages between participants were not permitted; instead all discussions were encouraged for the entire SM group.

### Demographic information

Information pertaining to age, self-reported race, gender, SLE disease duration, and SLE disease activity was recorded. SLE disease activity was measured with a Systemic Lupus Erythematosus Activity Questionnaire (SLAQ) at T0. The SLAQ is a validated self-assessment of disease activity that can be scored via a patient questionnaire without a physical examination or lab assessment. Scores can range from 0 to 44 [13, 14].

#### Outcome measures

The primary outcomes of interest were measures of feasibility including recruitment, compliance, and drop out proportions.

The secondary outcomes of interest included adherence to prescribed hydroxychloroquine (HCQ). Initially we planned to examine adherence to all medications, but there was much variability in the medications prescribed without enough of any one medication, besides, HCQ, to be able to examine differences between subjects. The majority of the subjects were prescribed HCQ, so we examined adherence to HCQ alone for the primary outcome measure, and in all subjects for the secondary outcome measures. Adherence was measured by 1) medication possession ratio (MPR) and 2) the medication adherence self-report inventory (MASRI) [15–17]. MPR is a widely accepted objective measure of medication adherence in children and adults [18–20]. The MPR is reported as a percentage calculated as: (total prescription days of supply/ (last prescription date – the first prescription date)). For example, if a patient only filled a one-month prescription twice during 6 months, so only had 60 days of medications available during the 6 months, the MPR would be 33%. The MPR data was obtained by the research team by phone, with subject’s permission, from the patient’s pharmacy and was calculated for T0 and T1. In order to capture adequate data to calculate the MPR, refill data was acquired for 3 months prior to the beginning of the study (MPR T0) and for 3 months after T1 (MPR T1). A MPR of ≥0.8 is generally accepted as good compliance [21]. The MASRI is a 6-item Likert Scale questionnaire which has been validated in patients with SLE [2]. The MASRI was calculated from REDCap questionnaires at T0 and T1.

Additional secondary outcomes of interest included measures of stress, self-efficacy, quality of life, SOA, SOC, and empowerment. Stress was measured by the Perceived Severity of Stress Questionnaire (PSQ) [22]. Self-efficacy was measured using the Children’s Arthritis Self-Efficacy scale (CASE), an 11-item Likert scale measure which has been validated in children and adolescents with juvenile idiopathic arthritis and was modified slightly for SLE [23]. Quality of life was assessed using the validated Simple Measure of the Impact of Lupus Erythematosus in Youngsters (SMILEY) index [24]. Sense of agency (SOA) was measured via three Likert scale questions aimed to tap three core concepts of agency: *competence* (“Blogging… makes me feel I have control over my own voice”*)*, *assertiveness* (“…enables me to assert myself”), and *confidence* (“…makes me feel I have a distinct voice”) [8]. Sense of community (SOC) was measured using a 22-item scale [8, 9, 25]. SOC consists of feelings of belonging to the community, having influence on, and being influenced by, the community, being supported by the community while also supporting them; and feelings of shared emotional connection. Empowerment was assessed using a validated quantitative Likert scale tool that measured 1) “empowering outcomes” (Additional file 1) [26]. The empowerment processes examine the effect of the online forum in the intervention group. The empowerment outcomes are applicable to both the control and intervention groups.

#### Statistical analyses

Data are shown as means with ± s.d. Statistical analyses for demographic information between were performed using Student’s t-test for continuous measures (data reported as means ± s.d.) and Chi-square testing for ordinal measures. Student’s t test was also used to compare differences between empowering outcome means between groups. Matched-pairs testing was used for all other analyses of continuous measures over time in groups and data is reported as means ± SEM. A Pearson product-moment correlation coefficient was computed to assess the relationships between outcome measures. JMP 12 software was used for analyses (JMP®, Version *12*. SAS Institute Inc., Cary, NC, 1989–2007). A power analysis was not calculated as this was a pilot study.

## Results

### Feasibility results

**Fig. 1:**
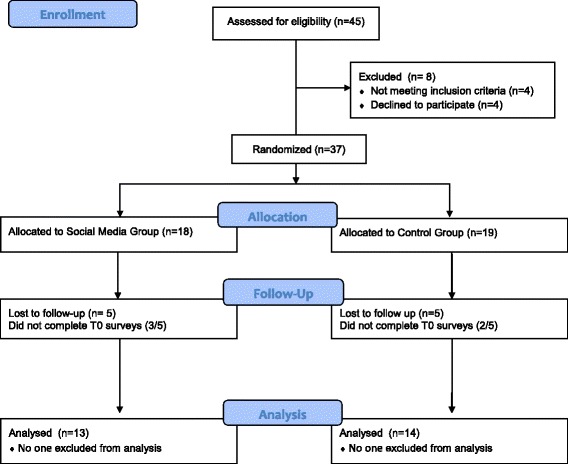
Flow Diagram for Participant Recruitment

Thirty seven subjects were randomized, with 27 completing the study (73%). Ten of the subjects were lost to follow up (27%). Once allocated to groups, 26% of the controls (5 of 19) and 28% of the SM subjects (5 of 18) were lost to follow up due to not completing all of the online questionnaires at T0 and/or T1. There was no statistical difference between the control and SM groups in regards to drop out (*p* = 0.8). Compliance was measured by the number of subjects who completed the online REDCap questionnaires at T0 or T1. 32 of the 37 subjects (86%) completed the first set of surveys at T0, and 27 of the 37 completed both T0 and T1 surveys (73%). Of the five subjects who did not complete T0 surveys, 2 were in the control group and 3 were in the SM group. (See Fig. 1). Mailers were sent to the ten subjects who were lost to follow up. Of the six responses that were returned, 4 subjects reported they were too busy and 2 stated that they forgot to check their email for the REDCap reminders. One subject suggested not using email in order to improve communication.

### Subject demographics

Thirty-seven subjects were recruited (19 controls and 18 SM subjects) with a total of 27 subjects completing the required surveys over time. (See Fig. 1).The mean age of the cohort was 18.1 ± 2.5 years, 96% were female, and the mean duration of disease (SLE) was 4.2 ± 2.6 years (Table 1). The racial composition of the cohort was primarily Caucasian and Hispanic. Five of the 10 subjects who were lost to follow up did not complete surveys at T0. Since demographic and SLE disease information was provided in the surveys at T0, comparisons of patient characteristics between those who completed the study and those who did not are not available. All of the subjects but 3 were taking HCQ throughout the study. One patient was diagnosed at entry into the study so no MPR data was available 3 months prior to that time (T0) and two subjects were prescribed chloroquine. These three subjects were excluded from the adherence analyses but the rest of their data was used for other secondary outcome analyses. The average number of medications per subject was 3.5 ± 2.4 per day. Only three of the subjects taking HCQ were not taking any other medications. The mean number of medications per day was not significantly associated with MPR at T0 (*p* = 0.35). The most common disease related medications other than anti-malarials were: prednisone (41%), mycophenolate mofetil (22%), angiotensin converting enzyme inhibitors (22%), and calcineurin inhibitors (7%).

**Table 1:** Demographic and population characteristics, stratified for the cohort and by intervention group (Social Media and Control) in systemic lupus subjects

	Total cohort (*n* = 27)		Control (*n* = 14)		Social Media (*n* = 13)		*p*-value*
Age (mean) yrs	18.1 ± 2.5		18.1 ± 2.5		18.2 ± 2.5		0.99**
Race	Cauc	13 (48%)	Cauc	5 (36%)	Cauc	8 (61%)	0.26
	AA	1 (4%)	AA	0 (0%)	AA	1 (8%)	
	Hispanic	10 (37%)	Hispanic	7 (50%)	Hispanic	3 (23%)	
	Asian	3 (11%)	Asian	2 (14%)	Asian	1 (8%)	
Gender (female)	26 (96%)		13 (93%)		13 (100%)		0.25
Disease duration (yrs)	4.2 ± 2.6		3.9 ± 2.4		4.5 ± 2.9		0.57**
SLAQ score	12.0 ± 8.5		10.3 ± 8.9		13.8 ± 8.1		0.31**
Current HCQ use	24 (89%)		12 (86%)		12 (92%)		0.59

*AA* African-American*, SLAQ* Systemic Lupus Erythematosus Activity Questionnaire*, HCQ* hydroxychloroquine, *p-value is for comparison between control group and social media group, **p-value calculated via Student t-test

Fourteen subjects were randomized into the control group and 13 into the SM group. There were no significant differences between groups for demographics or baseline disease activity (see Table 1). One subject in the SM group and two subjects in the Control group were not taking HCQ for the duration of the study period (*p* = 0.59).

### Secondary outcome measures

#### Medication adherence

In order to identify whether there was a change in medication adherence in the entire group from T0 to T1, MASRI and MPR were compared between these two time points. In order to evaluate those subjects whose MPR improved, subjects with a MPR < 1.0 (MPR of 1.0 = 100% adherence) were evaluated separately (See Table 2). The three subjects who were not on HCQ for the duration of the study included two controls and 1 SM subject. There were five controls and one SM subjects who had a MPR = 1 at T0. There was a significant improvement in the mean MPR in the entire cohort. This difference was also significant when the effect of those starting with a MPR = 1.0 were not included. The differences in MPR from T0 to T1 were examined to see whether there were any demographic characteristics associated with MPR improvement. There were no significant differences in change in MPR between age groups (*p* = 0.4) or race (*p* = 0.5).

**Table 2:** Pre- and Post-study medication adherence in entire cohort of systemic lupus subjects

	Pre-study (T0)	Post-study (T1)	*p*-value
MPR (*n* = 24)	0.77 ± 0.05	0.87 ± 0.03	***< 0.001*** *
MPR < 1.0 (*n* = 18)	0.69 ± 0.05	0.86 ± 0.04	< 0.001*
MPR ≥ 0.8	54% (13/24)	79% (19/24)	*0.07*
MASRI (*n* = 23)	86.6 ± 3.7	87.4 ± 3.9	0.46*
MASRI ≥ 0.8	76% (20/26)	81% (22/26)	0.48

*MPR* mediation possession ratio*, MASRI* medication adherence self-report inventory*, T0* prior to starting study*, T1* 6 weeks after study completion, Mean ± SEM, *calculated using paired t-test

The self-reported adherence (MASRI) was examined in those subjects for whom a MPR was calculated (1 subject did not report). The percentage of all subjects “adherent” (those with a MPR of ≥0.8, or 80%) improved from 54% to 79% (*p* = 0.07). The MASRI was universally higher than the MPR and did not significantly change over the course of the study. Interestingly, the self-reported adherence rate (≥80%) was 76% at T0 and 81% at T2, as compared to the objective measure of adherence (the MPR) of 54% and 79%, respectively. The correlation between MASRI and MPR was only moderate (*r* = 0.43 at T0 and *r* = 0.39 at T1), demonstrating a disconnect between subjective and objective adherence. There were no significant differences in MPR between time points with regard to race or age.

MPR and MASRI scores before and after the intervention were examined between the control and SM groups (Table 3). The mean MPR significantly improved from T0 to T1 in the SM group (0.75 ± 0.06 to 0.92 ± 0.03; *p* < 0.001), but not in the control group (0.79 ± 0.07 to 0.81 ± 0.05; *p* = 0.56). When the subjects with a baseline MPR of 1.0 were removed, the change in the mean MPR remained significant in the SM group (0.72 ± 0.05 vs. 0.93 ± 0.05; p < 0.001) and continued not to be significant in the control group (0.64 ± 0.07.21 vs. 0.76 ± 0.07; *p* = 0.08). The MASRI did not improve significantly over the course of the study.

**Table 3:** MPR and MASRI scores before and after intervention between control and social media participants

	Control			Social Media		
	T0 (n)	T1 (n)	*p*-value	T0 (n)	T1 (n)	*p*-value
MPR (mean)	0.79 ± 0.07 (12)	0.81 ± 0.05 (12)	0.56	0.75 ± 0.06 (12)	0.92 ± 0.03 (12)	< 0.001*
MPR < 1	0.64 ± 0.07 (7)	0.76 ± 0.07 (7)	0.08	0.72 ± 0.05 (11)	0.93 ± 0.05 (11)	< 0.001*
MPR ≥ 0.8	58% (7/12)	67% (8/12)	0.67	50% (6/12)	92% (11/12)	***0.03***
MASRI (mean)	87.8 ± 4.0 (12)	90.4 ± 2.4 (12)	0.76	85.4 ± 6.7 (11)	84.2 ± 7.7 (11)	0.44*
MASRI ≥0.8	79% (11/14)	93% (13/14)	0.28	75% (9/12)	75% (9/12)	1.0

*MPR* mediation possession ratio*, MASRI* medication adherence self-report inventory*, T0* prior to starting study*, T1* 6 weeks after study completion, Mean ± SEM, *calculated using paired t-test

Other secondary outcome measures, including measures of stress (PSQ), self-efficacy (CASE), and quality of life (SMILEY), were examined in all subjects before and after the intervention (Table 4). Analyses of scores were only included in subjects who completed the surveys at both time points. There were no significant changes in PSQ from T0 to T1 in the group as a whole, but the CASE (*p* = 0.05) and SMILEY (p = 0.05) scores neared significance. When secondary outcome measures were compared between the groups, the CASE was still significantly different in the SM groups only (*p* = 0.04), while the SMILEY was nearing significance in this group (*p* = 0.06). None of the measured secondary outcomes were different in the control group (Table 5).

**Table 4:** Secondary Outcome measures in entire cohort of SLE subjects

	Pre-study (T0)	Post-study (T1)	*p*-value
PSQ (*n* = 25)	0.49 ± 0.05	0.49 ± 0.05	0.94
CASE (*n* = 25)	36.4 ± 2.3	37.8 ± 2.0	**0.05**
SMILEY (*n* = 26)	63.2 ± 3.7	65.8 ± 3.7	**0.05**
SOA (*n* = 24)	16.6 ± 1.1	19.1 ± 1.0	0.08
SOC (*n* = 24)	140.8 ± 9.5	160.3 ± 7.6	**0.02**

Pre- and Post-study values expressed as Mean ± SEM

*Abbreviations: SLE* systemic lupus erythematosus*, T0* prior to starting study*, T1* 6 weeks after study completion*, PSQ* Perceived Severity of Stress Questionnaire*, CASE* Children’s Arthritis Self-Efficacy scale*, SMILEY* Simple Measure of the Impact of Lupus Erythematosus in Youngsters*, SOA* sense of agency*, SOC* sense of community

**Table 5:** Secondary Outcomes between Control and Social Media groups in SLE subjects

	Control			Social Media		
	T0 (*n* = 14)	T1 (*n* = 14)	*p*-value*	T0 (*n* = 12)	T1 (*n* = 12)	*p*-value*
PSQ	0.46 ± 0.06	0.50 ± 0.06	0.15	0.51 ± 0.07	0.48 ± 0.07	0.35
CASE	37.0 ± 2.9	36.6 ± 2.9	0.47	34.3 ± 3.4	38.5 ± 3.4	***0.04***
SMILEY	64.9 ± 5.4	67.0 ± 5.4	0.3	61.3 ± 5.0	64.4 ± 5.0	0.06
SOA	16.2 ± 1.5	17.3 ± 1.2	0.2	17.0 ± 1.8	20.8 ± 1.3	***0.03***
SOC	143.1 ± 14.0	152.2 ± 11.3	0.4	138.3 ± 13.4	168.3 ± 10.2	***0.03***

T0 and T1 values expressed as Mean ± SEM, *calculated using paired t-test*, T0* prior to starting study*, T1* 6 weeks after study completion

*Abbreviations*: *SLE* systemic lupus erythematosus, *PSQ* Perceived Severity of Stress Questionnaire, *CASE* Children’s Arthritis Self-Efficacy scale, *SMILEY* Simple Measure of the Impact of Lupus Erythematosus in Youngsters, *SOA* Sense of agency, *SOC* Sense of community

Although SOA and SOC improved in all subjects (*p* = 0.02), the improvement was only statistically significant in the SM group (Table 5). Two of the control subjects did not complete the T1 SOA and SOC surveys and therefore were not included in the analyses. The total mean score was significantly higher in the SM group (*p* = 0.04). In addition, mean scores for all seven empowering outcomes were higher in the SM group vs. the control group (Table 6). We were interested in potential explanations for the improvement in MPR in the SM group. In the SM group, there was a strong, positive correlation between MPR and SOA at T1 (*r* = 0.70; *p* = 0.01), but not at T0. There was a moderate positive correlation between MPR and SOC at T1 (*r* = 0.53; *p* = 0.08) but not at T0, suggesting that the impact of participating in the SM group may improve SOC and SOC. There was a strong, positive correlation between empowering outcome total and SOC (*r* = 0.77; *p* = 0.005) and SOA (*r* = 70; *p* = 0.02) but not with MPR (*r* = 0.26; *p* = 0.36).

**Table 6:** Empowerment Outcomes (T1) in Control and Social Media groups in SLE subjects

	Control (n = 13)	Social Media (n = 12)	*p*-value*
Feeling better informed	3.92 ± 0.39	3.96 ± 0.51	0.85
Increased confidence in physician relationship	3.96 ± 0.43	4.26 ± 0.49	0.11
Improved acceptance of illness	3.15 ± 0.51	3.65 ± 0.53	***0.03***
Increased confidence with treatment	3. 83 ± 0.49	3.90 ± 0.46	0.72
Increased optimism and control over the future	3.81 ± 0.55	4.27 ± 0.47	***0.03***
Increased self esteem	3.28 ± 0.86	3.63 ± 0.81	0.30
Improved social well being	2.69 ± 0.90	3.46 ± 0.81	***0.04***
Empowering Outcome Total Score	24.6 ± 2.54	27.1 ± 3.1	***0.04***

*Abbreviations: SLE* systemic lupus erythematosus

*p-value calculated via Student t-test

## Discussion

This pilot study is the first to create an online website created specifically to educate adolescents and young adults with SLE and to examine the effects of whether participation in a social media interaction with SLE peers improves outcomes. We were able to demonstrate that sustained compliance to complete the study was 73% versus 86% for the completing the first set of surveys only. We have demonstrated feasibility for developing the educational website, recruiting subjects, and identifying differences in medication adherence that appear to have been affected by being involved in a social media intervention.

We found a significant improvement in medication adherence for HCQ in adolescents and young adults with SLE using an online educational intervention that was enhanced by additional involvement in a social media intervention. To date, there are no prior studies that we know of whose aim is to improve medication adherence in SLE patients in this age group. The mean MPR of our cohort as a whole was 0.77 ± 0.23 (corresponding to 77%) before the intervention. This MPR was quite high at baseline compared to reports from the adult SLE literature. Koneru et al. found that 51% of 55 adult SLE patients were non-adherent to hydroxychloroquine using the MPR [2]. In order to net out a ceiling effect, we examined the change in scores for subjects who did not already have 100% adherence. In this group, participation in the study resulted in an improvement in MPR from 69% to 93%, with the SM group benefitting more than the educational only group (72% to 93% vs. 64% to 75%). The proportion of subjects who were adherent, defined as a MPR ≥ 80%, improved from 54% before the study to 79% after the study, with the SM group having a greater improvement than controls.

Our study capitalized on the online and social media skills that adolescents and young adults excel in. Other studies have utilized online internet-based self-management interventions in children. A review by Stinson et al. examined the literature for randomized controlled trials for online interventional studies with measurable outcomes in children [27]. None of the studies were performed in patients with rheumatologic conditions, nor did they utilize social media interventions. The majority utilized a health coach or therapist to tailor the information and strategies to individual needs. In varying degrees, the studies demonstrated improvements in health outcomes, disease-related knowledge, and quality of life. In a meta-analysis of 22 adult studies compared web-based self-management interventions to non-web based studies, internet interventions were associated with significantly improved health outcomes as compared to those that were non-web based [28]. A recent study examined the relationships between identity and medication use among young people with juvenile arthritis. Participants recognized that taking medication had both positive and negative consequences which had an impact both personally and socially. They note that increasing responsibility of taking their medications was empowering [29]. Stinson et al. investigated the patient reported self-management needs and acceptability of potential Web-based programs for self-management in adolescents with juvenile idiopathic arthritis. All of the respondents thought that a Web-based approach to interventions would be a good way to overcome barriers they perceived in accessing self-management information and skills [30]. Many of the components which were identified in this study as being helpful were addressed as topics that were part of our online educational program, www.facinglupustogether.com [11].

**Fig. 2:**
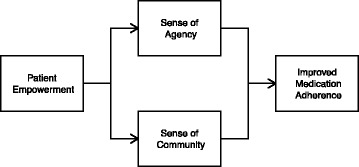
Proposed Mechanism for Improved Medication Adherence Using Social Media and Education

Secondary outcome measures that may contribute to the improvement in medication adherence in the SM group included SOA and SOC, as both of these showed a positive correlation with MPR. In addition, the total empowering outcome score was strongly correlated with SOA and SOC, suggesting that empowering outcomes had an indirect positive effect on improvement of medication adherence. While this correlation may not imply causality, it does suggest that there is a relationship between these variables. Empowerment impacts SOC and SOA, which in turn impacts medication adherence (see Fig. 2).

Empowerment is described as a “social process of recognizing, promoting, and enhancing people’s abilities to meet their own needs, solve their own problems, and mobilize necessary resources to take control of their own lives”. It is a process as well as a consequence [31]. Patient empowerment may start with education but continues as an active engagement in health care decisions. Online patient support groups have been shown to improve empowering outcomes in adults with various health conditions [32–34]. In the disease management of children, family empowerment has been shown to improve objective measures of disease control, such as pulmonary function tests in patients with asthma [35]. Parents were allowed to see the online educational modules in our study, but were encouraged not to participate in their children’s experience so we could be sure outcomes were based solely on the part of the subject. Future studies could include both child and parent involvement.

Assessments of medication adherence and self-management skills are certainly easier using self-reported measures. In our cohort, the self-reported adherence of the group was correlated only moderately with objective adherence measurement (the MPR). The percent of subjects with a self-report of being adherent (≥ 80%) at T0 was 77% for the entire group as compared to the objective measure of adherence (the MPR) of 54%. Self-reported adherence is not always consistent with, and usually lower than, objective measures of adherence as demonstrated in both adults and children in a variety of medical conditions [36–40]. This information suggests that objective measures of adherence, such as the MPR, should be utilized in children and young adults with SLE, instead of self-reported compliance.

Limitations of this study include the small sample size as well as that we measured medication adherence only to HCQ. Our initial goal was to examine adherence to all medications, but given the low total number of participants, it was not possible to compare adherence rates of different medications among the participants. Medication adherence has previously been shown to differ between drugs taken by adult SLE patients [41]. We plan to examine adherence rates to all SLE specific medications, including corticosteroids in our larger future study.

Future studies could utilize creative methods to remind participants to complete the study, such as texts and push notifications via cell phone [42]. The SMILEY index was developed for children ≤ age 18. We did not predict that our mean age would be as high as it was and we will limit recruitment to less than 18 years of age in the larger multi-site trial. Other measures used in the study have not been validated in children, including the SLAQ score. Future studies utilizing a self-reported SLAQ to assess disease activity may be more appropriately completed by the parents of subjects <age 18 years. We also plan to ask participants in the future if there were any significant disease flares during the study period, need for high dose steroids, hospitalizations, etc. that could affect the SLAQ score or other outcome measures.

The use of the MPR to assess medication adherence hinges on the assumption that subjects are taking a medication that they have filled. While this is a limitation, MPR is one of the most widely accepted methods of medication adherence that does not require pill counts or measuring drug levels (38). It is possible that the MPR at T1 may have been increased because patients/ families were more aware of the need to fill them. This potential bias would not explain any intergroup differences between the control and SM groups though.

Overall, the results of this study provide evidence for the feasibility of a large multi-site study examining the effect of a Web-based educational intervention with social media support in adolescents and young adults with SLE. We plan to develop a sustainable model in which these patients can gain self-management skills utilizing online education and peer support from social media. Future studies will focus on empowerment, sense of agency and sense of community as key concepts impacting the effect of these interventions on this patient population.

## Conclusions

This pilot study demonstrates the feasibility of a larger multi-site trial examining the effects of online education and social media support to improve medication adherence in young adults with SLE. All subjects had a significant improvement in medication adherence that was augmented if they also had social media support. Empowerment, sense of agency and sense of community appear to play key roles in the possible mechanisms of this improvement.

## Additional file

Additional file 1: Empowering Outcomes. (PDF 464 kb)

## Abbreviations

CASE: Children’s Arthritis Self-Efficacy scale
HCQ: Hydroxychloroquine
MASRI: Medication adherence self-report inventory
MPR: Medication possession ratio
PSQ: Perceived Severity of Stress Questionnaire
REDCap: Research electronic data capture
SLAQ: Systemic Lupus Erythematosus Activity Questionnaire
SLE: Systemic lupus erythematosus
SM: Social media
SMILEY: Simple Measure of the Impact of Lupus Erythematosus in Youngsters
SOA: Sense of agency
SOC: Sense of community
