# Supplementary material for: Improvement of medication adherence in adolescents and young adults with SLE using web-based education with and without a social media intervention, a pilot study
Source: Pediatr Rheumatol Online J. 2018 Mar 14;16:18. doi: 10.1186/s12969-018-0232-2 (PMC5852975; doi:10.1186/s12969-018-0232-2)
Supplement: Supplementary file 1 — Empowering Outcomes. (PDF 464 kb) [file 12969_2018_232_MOESM1_ESM.pdf]

Empowering Outcomes

Answer categories:(1) completely disagree; (2) disagree; (3) neither agree nor disagree, (4) agree, (5) completely agree

	Completely Disagree	Disagree	Neither Agree Disagree	Agree	Completely Agree
**Being Better Informed**					
**Through my participation in the Facing Lupus Together Project**					
I feel better informed as a patient.	❑	❑	❑	❑	❑
I understand my illness better.	❑	❑	❑	❑	❑
I have a clearer picture about my illness.	❑	❑	❑	❑	❑
I feel like I have more (correct) knowledge at my disposal to deal better with my illness.	❑	❑	❑	❑	❑
**Feeling More Confident in the Relationship with Their Physician**					
**Through my participation in the Facing Lupus Together Project**					
I feel better prepared for a doctor's appointment.	❑	❑	❑	❑	❑
I am more knowledgeable about which questions to ask my physician	❑	❑	❑	❑	❑
I can explain my needs to my physician better.	❑	❑	❑	❑	❑
I have more courage to raise matters with my physician.	❑	❑	❑	❑	❑
I am more able to oppose my physician better.	❑	❑	❑	❑	❑
the relationship with my physician has improved.	❑	❑	❑	❑	❑
the relationship with my physician has deteriorated.	❑	❑	❑	❑	❑
I am more able to judge when I really need the help of my physician	❑	❑	❑	❑	❑
I feel less dependent on my physician.	❑	❑	❑	❑	❑
I am more able to think along with my physician about my treatment.	❑	❑	❑	❑	❑
**Improved Acceptance of the Illness**					
**Through my participation in the Facing Lupus Together Project**					
I am able to be more open about my own illness.	❑	❑	❑	❑	❑
I can tell others more easily when I am no longer able to do something	❑	❑	❑	❑	❑

	I can ask others for help more quickly.	❑	❑	❑	❑	❑
	I can give in to my illness better.	❑	❑	❑	❑	❑
	I can accept my illness better.	❑	❑	❑	❑	❑
**Feeling More Confident about the Treatment**						
**Through my participation in the Facing Lupus Together Project**						
	I can stick to my treatment regime better.	❑	❑	❑	❑	❑
	I am more able to follow the medical guidelines and advice of my physician	❑	❑	❑	❑	❑
	I know where to go with questions about my illness.	❑	❑	❑	❑	❑
	I feel I am more skilled at dealing well with my illness.	❑	❑	❑	❑	❑
	I feel able to make the right decisions with regard to my illness.	❑	❑	❑	❑	❑
**Increased Optimism and Control over the Future**						
**Through my participation in the Facing Lupus Together Project**						
	I feel more in charge of the course of my illness.	❑	❑	❑	❑	❑
	I feel I can influence my illness myself.	❑	❑	❑	❑	❑
	I feel more in control over what is happening to me.	❑	❑	❑	❑	❑
	I feel less in control over what is happening to me.	❑	❑	❑	❑	❑
	I feel that what happens to me in the future is to a large degree dependent on myself	❑	❑	❑	❑	❑
	I have learned to be more positive.	❑	❑	❑	❑	❑
	I have more faith in the future.	❑	❑	❑	❑	❑
	I have less faith in the future.	❑	❑	❑	❑	❑
**Enhanced Self-Esteem**						
**Through my participation in the Facing Lupus Together Project**						
	I have a greater sense of worth.	❑	❑	❑	❑	❑
	I have a more positive attitude towards myself.	❑	❑	❑	❑	❑
	I am in general more content with myself.	❑	❑	❑	❑	❑
**Enhanced Social Well-Being**						
**Through my participation in the Facing Lupus Together Project**						
	I feel less lonely.	❑	❑	❑	❑	❑
	I have made new social contacts.	❑	❑	❑	❑	❑
